# S02-1: Improving Physical Activity policies and their impact on health Equity: An overview of the IMPAQT project

**DOI:** 10.1093/eurpub/ckae114.202

**Published:** 2024-09-26

**Authors:** Catherine Woods, Kevin Volf, Aurelie van Hoye, Peter Gelius, Sven Messing, Sarah Forberger, Joanna Zukowska, Romanika Okraszewska, Nicole den Braver, Jeroen Lakerveld, Fleur Heuvelman, Petru Sandu, Sven Messing, Migle Baceviciene, Rasa Jankauskiene

**Affiliations:** Physical Activity for Health Research Centre (PAfH), Department of Physical Education and Sport Sciences, Faculty of Education and Health Sciences, University of Limerick, Ireland; Physical Activity for Health Research Centre (PAfH), Department of Physical Education and Sport Sciences, Faculty of Education and Health Sciences, University of Limerick, Ireland; Physical Activity for Health Research Centre (PAfH), Department of Physical Education and Sport Sciences, Faculty of Education and Health Sciences, University of Limerick, Ireland; Institute of Sport Sciences, Université de Lausanne, Switzerland; WHO Collaborating Centre for PA and Public Health, Department of Sport Science and Sport, Friedrich-Alexander-Universität Erlangen-Nürnberg FAU Erlangen-Nürnberg, Germany; Leibniz-Institute for Prevention Research and Epidemiology – BIPS, Germany; Faculty of Civil & Environmental Engineering, Department of Transportation, Gdansk University of Technology, GUT, Poland; Faculty of Civil & Environmental Engineering, Department of Transportation, Gdansk University of Technology, GUT, Poland; Amsterdam University Medical Centre, Netherlands; Amsterdam University Medical Centre, Netherlands; Amsterdam University Medical Centre, Netherlands; University Babes-Bolyai, Cluj-Napoca, Romania; University Babes-Bolyai, Cluj-Napoca, Romania; Klaipeda’s University, Faculty of Health Sciences, Health Research and Innovation Science Centre; Klaipeda’s University, Faculty of Health Sciences, Health Research and Innovation Science Centre

## Abstract

**Purpose:**

The growing burden of non-communicable diseases (NCDs) requires effective public policy to change systems instead of individuals and create supportive contexts that reduce NCDs and health inequity. A major determinant of disease reduction is physical activity (PA). Many countries have policies in place, but the implementation of those policies is unknown. This presentation will show how Germany, Ireland, Lithuania, Netherlands, Poland and Romania are collaborating to gather data to benchmark the extent of implementation of their PA policies and their health equity impact.

**Methods:**

An innovative tool, the PA-Environment Policy Index for Equity (PA-EPIQ) will be developed and used to test and promote policy benchmarking as a tool to improve policy implementation in six countries. This will involve mixed methods including evidence synthesis, document analysis and case reports to ‘evidence ground’ countries’ policy implementation status in relevant domains, e.g. health, sport, education, transport. Quantitative and qualitative evidence validation with policymakers will ensure the comprehensive representation of government action. While engagement with stakeholders – both experts and citizens – from each country will independently assess the evidence on their governments’ action, comparing it to international best practice.

**Results:**

IMPAQT will generate six country-specific policy index scores, which will be translated into report cards containing critical implementation gaps and policy recommendations for each country. It will also bring the individual country reports together and create a unified report for citizens, scientific communities, and policymakers.

**Conclusions:**

IMPAQT is based on scientific research as well as the involvement of policymakers, experts and citizens through a co-production approach in 6 EU countries. Transnational comparisons will provide international benchmarks to support policymakers in enhancing health equity in PA policy development.

**Support/Funding Source:**

ERA4Health, GA N° 101095426 of the EU Horizon Europe Research and Innovation Programme

